# Preparation of *Siraitia grosvenorii* Leaf-Derived Carbon Dots and Its Effect on Tissue Culture Seedlings of *Siraitia grosvenorii*

**DOI:** 10.3390/nano16181185

**Published:** 2026-09-20

**Authors:** Lizhen Zhang, Yihan Liu, Dongye Yang, Jiayue Deng

**Affiliations:** 1 School of Chemistry and Life Health, Guilin Normal College, Guilin 541119, China; zhanglizhen@mail.glnc.edu.cn (L.Z.);; 2 Suzhou Institute of Pharmaceutical Innovation, Suzhou Industrial Park, Suzhou 215218, China; 3 Scientific Research Center, Guilin Medical University, Guilin 541000, China

**Keywords:** *Siraitia grosvenorii*, carbon dots, tissue culture, adventitious buds

## Abstract

In order to investigate the effects of carbon dots on *Siraitia grosvenorii* tissue culture, fluorescent carbon dots (L-CDs) were prepared via a one-step hydrothermal method using *S. grosvenorii* leaves as a novel carbon source. The synthesized L-CDs exhibited blue fluorescence under UV irradiation, with an optimal excitation wavelength of 379 nm and an optimal emission wavelength of 451 nm. The L-CDs demonstrated excellent biocompatibility, superior anti-photobleaching and salt-tolerance properties, and high stability across a wide pH range of 1–12, making them highly suitable for plant tissue culture. Notably, 0.6 mg·L^−1^ L-CDs significantly promoted the growth of *S. grosvenorii* tissue-cultured seedlings and increased their propagation coefficient. Further studies revealed that 0.6 mg·L^−1^ L-CDs significantly enhanced antioxidant enzyme activities, with SOD, POD, and CAT activities increasing by 82.61%, 33.33%, and 58.82%, respectively, compared to the control group. This study highlights the potential of L-CDs as highly efficient and non-toxic growth promoters in plant tissue culture, providing a sustainable and effective strategy for their application in plant biotechnology.

## 1. Introduction

*Siraitia grosvenorii*, one of the first batch of medicinal and edible homologous species published in China and a genuine medicinal herb among Guangxi’s “Ten Authentic Herbs of Guangxi (Gui Shi Wei)”, boasts high nutritional value and prominent therapeutic effects, including relieving cough, eliminating phlegm, moistening the lungs, lowering blood sugar, protecting the liver, and exerting anti-cancer effects [1,2]. The fruit contains a unique active ingredient, mogroside V, which is characterized by high sweetness, low caloric content, safety, non-toxicity, and excellent taste. It serves as an ideal natural sweetener and an optimal health supplement for patients with obesity, hypertension, diabetes, and heart disease. With the implementation of the Healthy China 2030 Planning Outline, the concepts of healthy diet and wellness-oriented elderly care have gradually taken root in people’s minds. Against this backdrop, *S. grosvenorii*, which possesses both medicinal and nutritional values, is upgrading from a “raw material supplier” to a producer of “functional foods and general health products,” thereby ushering in a golden era of development. However, the relatively low content of mogroside in fresh fruits (approximately 0.45%) has long plagued the development of this industry due to excessively high raw material costs. There is an urgent need to cultivate superior varieties and improve planting efficiency to overcome this bottleneck [3]. Currently, the *S. grosvenorii* seedling industry has fully entered a technology-empowered stage characterized by virus-free tissue-cultured seedlings, diversified varieties, standardized production, and scaled operations. However, it simultaneously faces challenges such as uneven market quality, the iteration of new varieties, and cost control.

Nanotechnology, one of the most significant scientific fields in the modern era, has achieved comprehensive interdisciplinary integration. Nanoscale particles possess unique physicochemical properties and exert significant effects on in vitro plant growth and development. They have demonstrated positive roles in various processes, including plant micropropagation, callus induction, somatic embryogenesis, cell suspension culture, and plant sterilization [4].

Carbon dots (CDs), a novel class of carbon nanomaterials, possess remarkable advantages such as excellent photostability, good biocompatibility, low cytotoxicity, and facile surface functionalization. They have demonstrated broad application prospects in various fields, including bioimaging [5,6], chemical sensing [7,8], environmental monitoring [9,10], disease diagnosis [11,12], and photocatalysis [13,14]. Biomass, rich in renewable, non-toxic, and eco-friendly carbon sources such as cellulose, hemicellulose, lignin, sugars, and proteins, serves as an ideal precursor for the preparation of carbon dots. Biomass-derived carbon dots feature abundant raw materials, low cost, environmental friendliness, and facile scalability, demonstrating significant application potential across multiple fields [15,16]. Studies have shown that carbon dots derived from marine alga Hizikia fusiformis can be utilized for fluorescent bioimaging and promote rice seedling growth [17]. Carbon-based nanomaterials can serve as growth promoters in plant tissue culture systems by enhancing the photosynthetic rate and gene expression, thereby improving callus formation and regeneration while reducing the demand for chemical growth regulators [18]. It has been reported that carbon dots prepared via a microwave-assisted method, using fresh tomatoes as a carbon source and amino acids as dopants, can serve as highly efficient and non-toxic growth promoters to improve plant morphogenesis and proliferation rates [19]. Using bee pollen waste as a raw material, two types of biomass carbon dots (WBCDs and EBCDs) were successfully prepared via a one-step hydrothermal method. They exhibited excellent sensing performance towards Fe^3+^ and good biocompatibility for cell imaging. Furthermore, they significantly promoted lateral root growth in *Arabidopsis thaliana*, demonstrating great potential for development as novel plant growth regulators [20]. However, to date, no studies have investigated the application of *S. grosvenorii*-derived carbon dots in its own tissue culture. The green synthesis of carbon dots from waste biomass and the expansion of their applications in agricultural production hold significant research implications.

**Figure 1:**
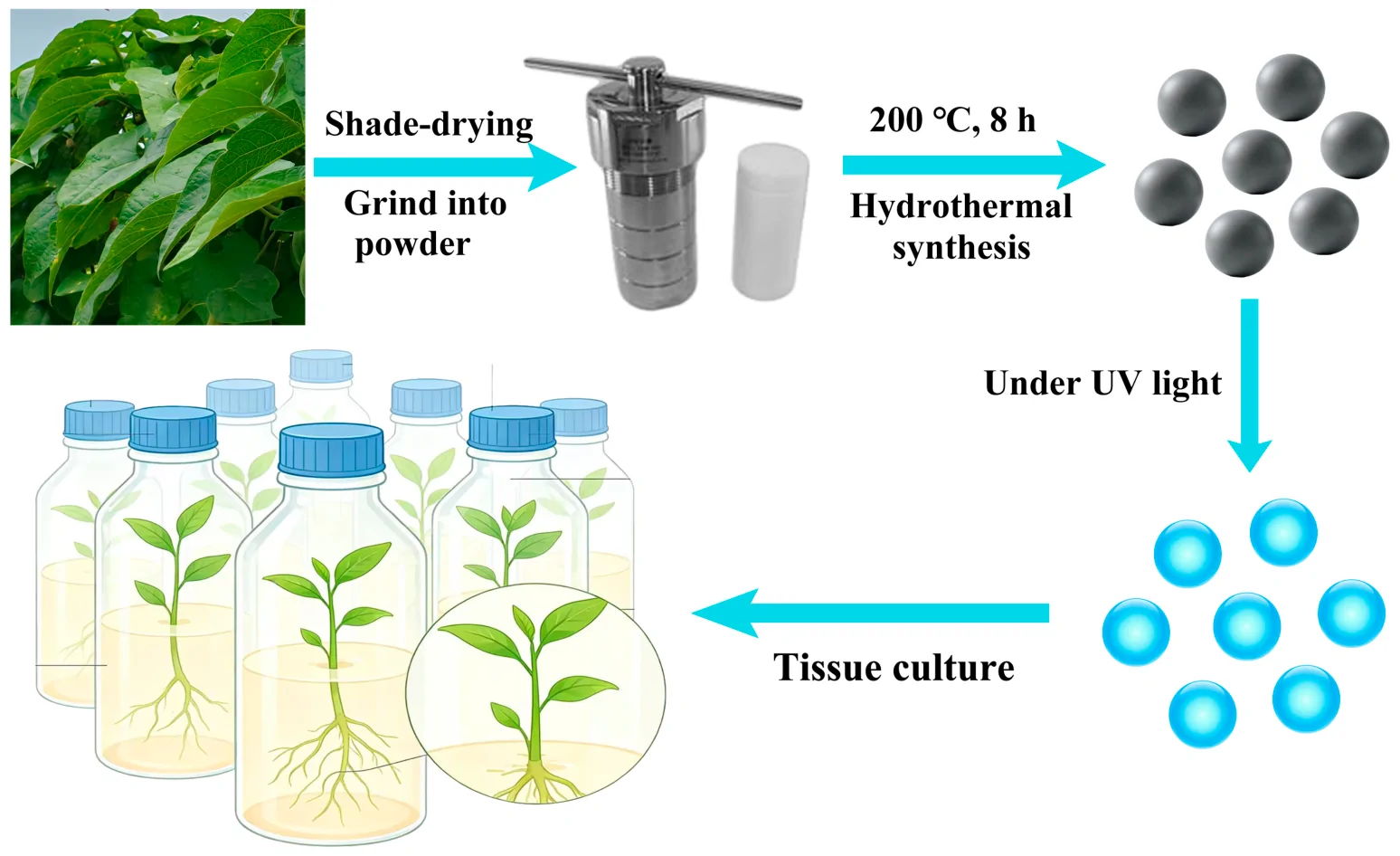
Schematic illustration for the preparation of L-CDs and their tissue culture applications.

In this study, *S. grosvenorii* leaf-derived carbon dots (L-CDs) were synthesized via a one-step hydrothermal method using *S. grosvenorii* leaves as precursors (Figure 1). By leveraging the unique properties of these carbon dots, we investigated their effects on *S. grosvenorii* tissue-cultured plantlets. This work not only expands the application of carbon dots in the field of plant tissue culture but also provides novel methods for *S. grosvenorii* seedling cultivation and new insights for agricultural development.

## 2. Materials and Methods

### 2.1. Materials

Agar powder was purchased from Guangdong Huankai Microbial Sci-Tech Co., Ltd. (Guangdong, China); MS medium was obtained from Qingdao Hi-Tech Industrial Park Hope Bio-Technology Co., Ltd. (Qingdao, China); Analytical-grade plant growth regulators, such as 6-benzylaminopurine, were purchased from Shanghai Jizhi Biochemical Technology Co., Ltd. (Shanghai, China); Catalase (CAT), superoxide dismutase (SOD), and peroxidase (POD) ELISA kits were obtained from Wuhan GeneMei Biotechnology Co., Ltd. (Wuhan, China); The leaves of *S. grosvenorii* were provided by the Guangxi Institute of Botany, Guangxi Zhuang Autonomous Region, Chinese Academy of Sciences.

### 2.2. Preparation of L-CDs

The naturally air-dried leaves of *S. grosvenorii* were ground into powder by a Chinese herbal medicine grinder and sieved through a 125 μm mesh. Accurately weigh 10.0 g of *S. grosvenorii* leaf powder, add 100 mL of distilled water, and magnetically stir for 10 min to ensure thorough mixing. The mixture was then transferred to a polytetrafluoroethylene (PTFE) autoclave and heated at 200 °C for 8 h in an electric blast drying oven. After cooling to room temperature, the mixture was filtered through a 0.22 μm microporous membrane to obtain a brownish-yellow solution containing the *S. grosvenorii* leaf-derived carbon dots (L-CDs). The resulting solution was then dialyzed against a cellulose dialysis membrane (MWCO: 500 Da) for 24 h to remove excess precursors and generate small molecules. Subsequently, the dialyzed solution was lyophilized (freeze-dried) for 3 days to yield the L-CDs powder. Finally, the lyophilized powder was dissolved in deionized water to prepare a stock solution, and the pH of the solution was adjusted to 7.0 prior to use.

### 2.3. Characterization

The particle size and morphology of the L-CDs were characterized by transmission electron microscopy (TEM, JEOL-JEM-2100 F, Tokyo, Japan). The chemical composition and structure were analyzed using Raman spectroscopy (LabRAM HR Evolution, HORIBA, Tokyo, Japan), with a 785 nm argon ion laser as the excitation source. X-ray photoelectron spectroscopy (XPS, Thermo Fisher Scientific, Waltham, MA, USA), and Fourier transform infrared spectroscopy (FT-IR, Perkin-Elmerls-55, Waltham, MA, USA). Furthermore, the optical properties of the L-CDs were investigated via UV-vis absorption (Specord^®^, 200plus, Jena, Germany) and fluorescence spectroscopy (FL, F-4600, Hitachi, Tokyo, Japan).

### 2.4. Fluorescence Spectra Fluorescence Quantum Yield of L-CDs

The fluorescence spectra were recorded on a fluorescence spectrophotometer (FL, F-4600, Hitachi, Tokyo, Japan). The excitation and emission slit widths were set at 5 nm. The L-CDs aqueous solution with a concentration of 0.05 mg·mL^−1^ was used for the measurements. The scan speed was 1200 nm/min.

Quinine sulfate (dissolved in 0.1 M H_2_SO_4_; QY = 0.54) was used as a reference standard. The absorbance of both L-CDs and quinine sulfate was measured via UV-Vis spectroscopy and maintained below 0.05. The fluorescence emission spectra of L-CDs and quinine sulfate were recorded in the range of 350–600 nm using a fluorescence spectrophotometer. The relative fluorescence quantum yield of L-CDs was then calculated according to the following equation:

Φ_S_ = Φ_R_ × (I_S_/I_R_) × (A_R_/A_S_) × (η_S_^2^/η_R_^2^) (1)

where Φ is the fluorescence quantum yield, I is the fluorescence integrated area at 350 nm excitation wavelength, A is the absorbance, and η is the refractive index. S and R represent the L-CDs and quinine sulfate to be measured, respectively. The concentration of L-CDs and the reference standard solution to be tested is very low in this experiment, so the effect of the different solutes can be ignored.

### 2.5. Biocompatibility Assessment of L-CDs

L929 cells were cultured in Dulbecco’s Modified Eagle Medium (DMEM) supplemented with 10% (*v*/*v*) fetal bovine serum (FBS) and 1% penicillin-streptomycin. The cells were maintained at 37 °C in a humidified atmosphere containing 5% CO_2_. L929 cells in the logarithmic growth phase with good viability were inoculated into 96-well plates at a density of 1 × 10^4^ cells per well in 180 μL of medium. After the cells reached confluence, the culture medium was replaced with fresh medium. Subsequently, 20 μL of L-CDs solution was added to achieve final concentrations of 0, 0.2, 0.4, 0.6, 0.8, and 1.0 mg·mL^−1^. The cells were then incubated at 37 °C in a humidified atmosphere containing 5% CO_2_ for 24 h. Subsequently, 20 μL of CCK-8 reagent was added to each well, and the cells were co-incubated for 2 h. The optical density (OD) values were then measured at 450 nm using a microplate reader. Cell viability was calculated according to the following formula (all experiments were performed in triplicate, and the mean values were recorded):

(2) cell viability%=average absorbance of treatedaverage absorbance of control ×100%

### 2.6. In Vitro Cultivation of S. grosvenorii Tissue-Cultured Seedlings

Plump and well-developed *S. grosvenorii* seeds were selected and their seed coats were removed. The seeds were then surface-sterilized by soaking in a 0.1% mercuric chloride (HgCl_2_) solution for 10 min, washed 3–4 times with sterile distilled water, and soaked in sterile water for 30 min. Finally, the seeds were inoculated onto basal MS medium. The inoculated *S. grosvenorii* seeds were placed in a culture chamber and incubated in complete darkness. After germination, the seeds were cultured under light conditions at 25 ± 1 °C with a 16 h photoperiod and a light intensity of 2000 lx (the same hereafter). The growth of *S. grosvenorii* adventitious shoots was observed and recorded.

### 2.7. Effects of L-CDs on the Growth of S. grosvenorii Tissue-Cultured Seedlings

The *S. grosvenorii* tissue-cultured seedlings were subcultured on MS medium supplemented with 1.0 mg·L^−1^ 6-BA and 0.2 mg·L^−1^ IBA. Different concentrations of L-CDs (0, 0.3, 0.6, 0.9, and 1.2 mg·L^−1^) were added to the medium. The culture medium without L-CD serves as the blank control group. For each concentration, 30 shoots were randomly inoculated. After 20 days, the growth of the seedlings was monitored to investigate the effects of L-CDs on the growth of the tissue-cultured seedlings. All experiments were performed in triplicate, and the data are presented as the mean values.

### 2.8. Detection of Antioxidant Enzymes in S. grosvenorii Tissue-Cultured Seedlings

The *S. grosvenorii* tissue-cultured seedlings were collected, accurately weighed, and flash-frozen in liquid nitrogen before being ground into a fine powder. The activities of catalase (CAT), superoxide dismutase (SOD), and peroxidase (POD) were then determined using ELISA kits according to the manufacturer’s instructions. All experiments were performed in three biological replicates.

The content of malondialdehyde (MDA) was measured by the thiobarbituric acid (TBA) assay. A 5 g sample of *S. grosvenorii* tissue-cultured seedlings was homogenized in 10 mL of pre-chilled 5% trichloroacetic acid (TCA) in an ice bath. After centrifugation at 10,000 r/min for 10 min, 2 mL of the supernatant was collected and mixed with 2 mL of 0.5% thiobarbituric acid (TBA). The mixture was heated in a boiling water bath (100 °C) for 20 min and then rapidly cooled. Following another centrifugation at 10,000 r per minute for 10 min, the absorbance of the supernatant was measured at 450, 532, and 600 nm. All experiments were performed in three biological replicates (n = 3). The MDA concentration C (μmol·L^−1^) and content (nmol·g^−1^ FW) were calculated according to the following formula:

C (μ mol L^−1^) = 6.45 × (A_532_ − A_600_) − 0.56 × A_450_ (3)

MDA (n mol g^−1^) = (C × V × 1000)/W (4)

where C is the MDA concentration (μmol·L^−1^), V is the total volume of the extraction solution (mL), and W is the fresh weight of the sample (g).

### 2.9. Detection of the Physiological Characteristics in S. grosvenorii Tissue-Cultured Seedlings

The chlorophyll content was determined by the UV-Vis spectrophotometric method. Ribulose-1,5-bisphosphate carboxylase/oxygenase (RuBisCO) activity, was measured using a Wuhan Gene Beauty Biotechnology Co., Ltd. ELISA assay kit (Wuhan, China). The carbohydrate content was determined using the sulfuric sulfate-anthone method. The protein content was determined by a Kjeldahl automatic N analyzer (K9840, Hanon, Jinan, China).

### 2.10. Statistical Analysis

The data were presented as the mean ± standard deviation. A one-way analysis of variance (ANOVA) with the Fisher LSD test was used to analyze the variation among different treatments (Origin Statistics 2019b, *p* < 0.05).

## 3. Results

### 3.1. Morphological and Structural Characterization of L-CDs

**Figure 2:**
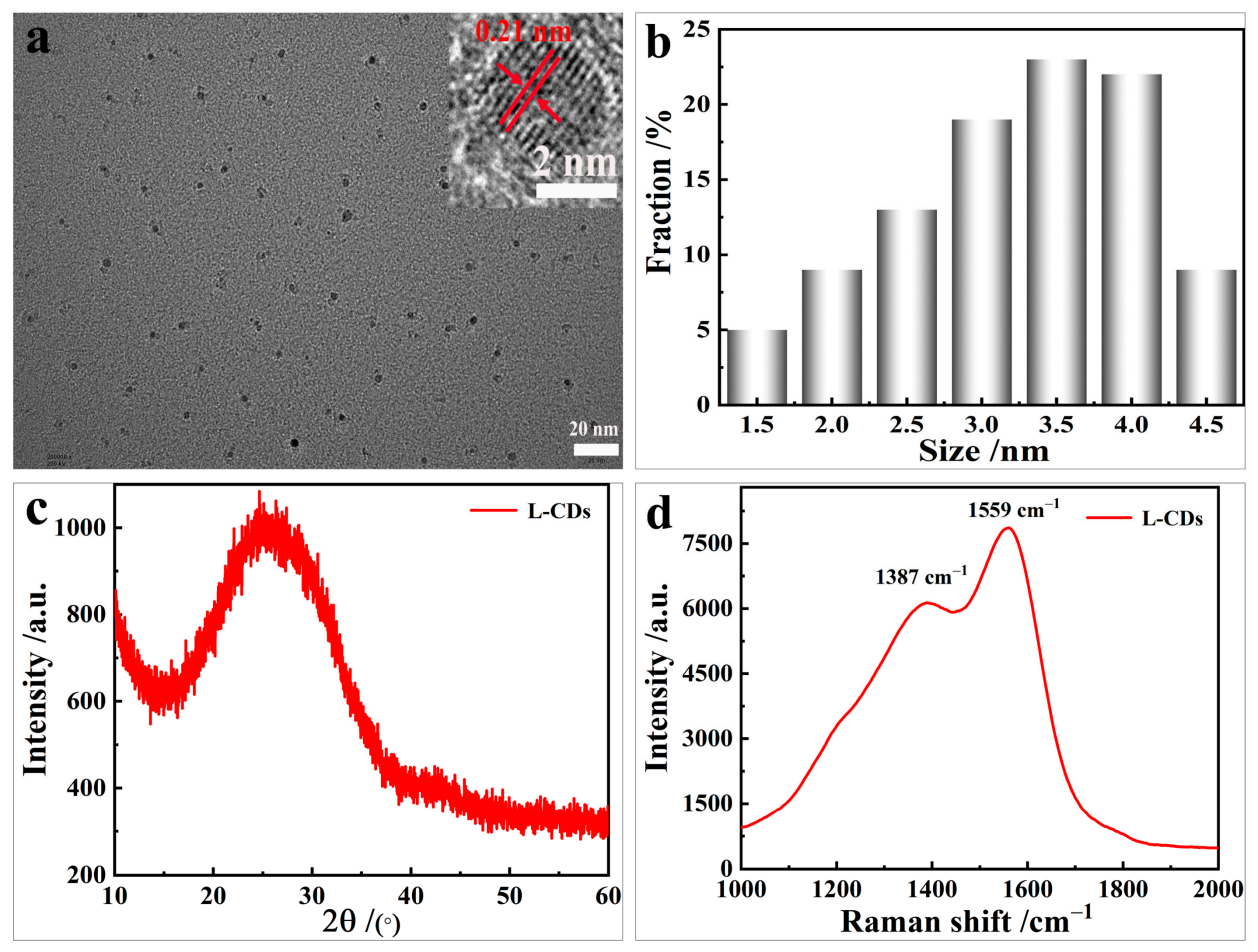
TEM image (**a**) and the corresponding size distribution (**b**) of L-CDs. (**c**) XRD and (**d**) Raman spectra of L-CDs.

A series of characterizations, including transmission electron microscopy (TEM), X-ray diffraction (XRD), Raman spectroscopy, Fourier transform infrared spectroscopy (FT-IR), and X-ray photoelectron spectroscopy (XPS), were conducted to investigate the morphology, structure, composition, and surface functional groups of the synthesized L-CDs. First, the morphology and particle size of the prepared L-CDs were characterized by TEM. As shown in Figure 2a, the L-CDs exhibited uniform, well-dispersed, nearly spherical particles with a particle size distribution of 1.5−4.5 nm (Figure 2b), indicating that the L-CDs consisted of nanodots with a relatively uniform size distribution. The high-resolution TEM (HRTEM) image in the inset of Figure 2a clearly reveals lattice fringes with a lattice spacing of 0.21 nm, which corresponds to the (100) lattice plane of graphitic carbon [21].

The X-ray diffraction pattern exhibited a broad peak centered at 25.4° (Figure 2c), corresponding to the (002) crystal plane of the graphitic carbon structure, which is attributed to a highly disordered graphitic carbon framework [22]. As shown in Figure 2d, the Raman spectrum of the L-CDs displayed two distinct characteristic peaks: the disorder-induced (D) band at 1387 cm^−1^ and the crystalline (G) band at 1559 cm^−1^, which are assigned to the sp^3^ and sp^2^ hybridized carbon atoms, respectively. The intensity ratio of the D band to the G band (ID/IG) is a crucial indicator for evaluating the defect density of carbon materials [23]. In this study, the ID/IG ratio of the prepared L-CDs was approximately 0.78, indicating the coexistence of both graphitic and amorphous carbon structures. These Raman spectroscopy results are consistent with the TEM and XRD characterization results.

**Figure 3:**
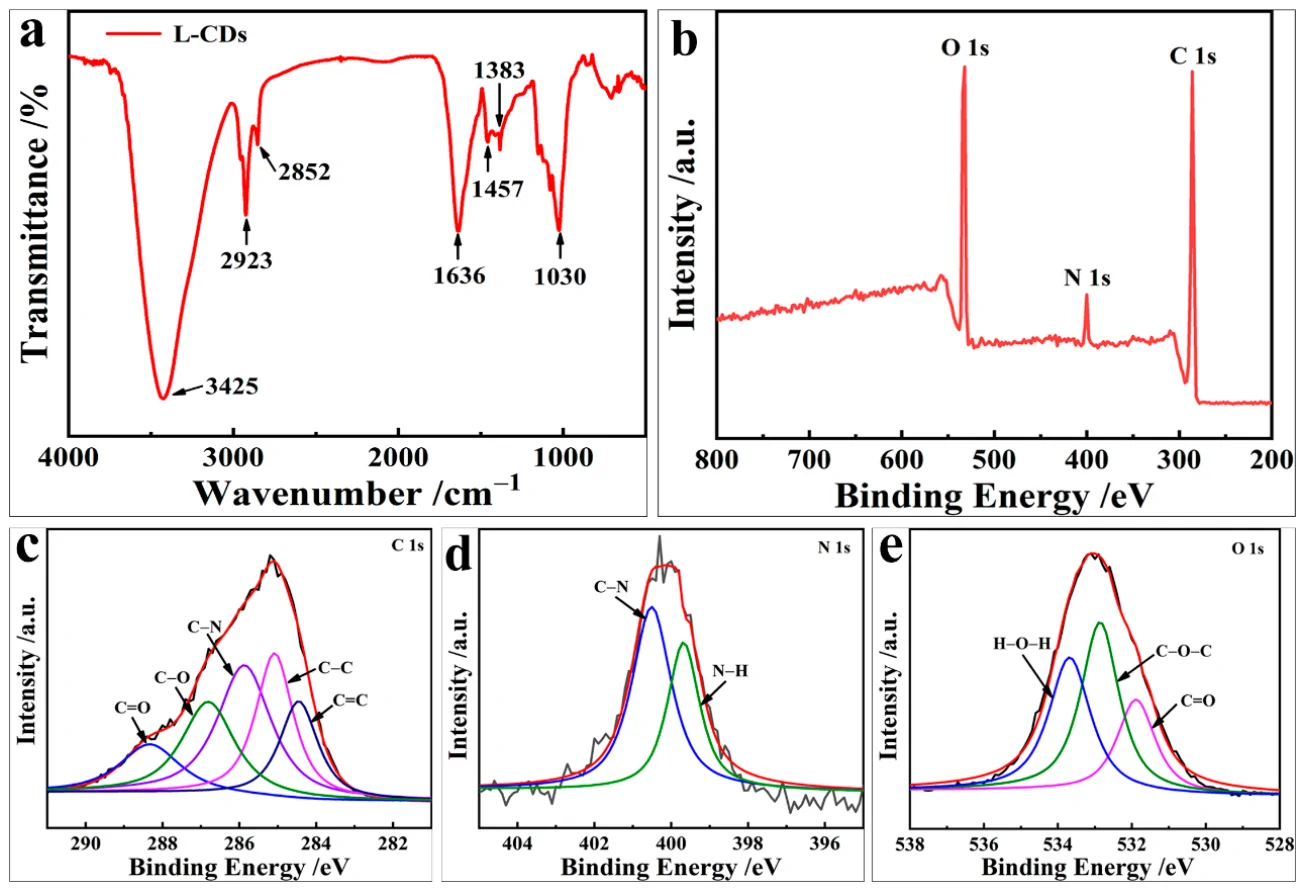
(**a**) FT-IR of L-CDs, (**b**) XPS survey spectra of L-CDs, (**c**–**e**) High-resolution C1s, N1s and O1s XPS spectra of L-CDs, respectively.

To further investigate the structure and surface functional groups of the synthesized L-CDs, Fourier transform infrared spectroscopy was performed. As shown in Figure 3a, the characteristic peak at 1030 cm^−1^ corresponds to the stretching vibration of C–O bonds, while the peaks at 1383 cm^−1^ and 1400 cm^−1^ are assigned to the stretching vibrations of C–N bonds [24]. The sharp peak at 1636 cm^−1^ is attributed to the C=O stretching vibration, and the peaks at 2852 cm^−1^ and 2923 cm^−1^ correspond to the stretching vibrations of aliphatic C–H bonds. Additionally, the peak located at 3425 cm^−1^ is associated with the stretching vibrations of O–H/N–H bonds [25]. These results indicate that the surface of the L-CDs is rich in oxygen and nitrogen containing functional groups, such as hydroxyl and amino groups. These hydrophilic functional groups can significantly enhance the dispersibility and stability of the L-CDs in aqueous solutions.

X-ray photoelectron spectroscopy was employed to analyze the surface state and elemental composition of the L-CDs. As shown in Figure 3b, the XPS survey spectrum exhibited three distinct characteristic peaks at 285.8 eV, 400.3 eV, and 532.4 eV, which were attributed to C1s, N1s, and O1s, respectively. The atomic percentages of C, N, and O are 69.23%, 4.45%, and 26.32%, respectively. The original spectrum was further deconvoluted using XPS Peak software (XPSpeak41). The high-resolution C1s spectrum (Figure 3c) could be fitted into five peaks: the peak at 288.3 eV was assigned to the C=O bond, 286.8 eV to the sp^3^ hybridized C–O bond, 285.8 eV to the sp^3^ hybridized C–N bond, 285.0 eV to the sp^3^ hybridized C–C bond, and 284.4 eV to the sp^2^ hybridized C=C bond [26]. The relative proportions of C=O, C-O, C-N, C-C, O-C=O, and C=C are 12.21%, 20.26%, 29.17%, 22.74%, and 15.62%, respectively. These results indicate that the L-CDs are primarily composed of graphitic carbon (sp^2^) and disordered carbon (sp^3^), and their surfaces are rich in hydrophilic functional groups, such as hydroxyl and carbonyl/carboxyl groups. The high-resolution N1s spectrum was deconvoluted into two Gaussian peaks (Figure 3d), corresponding to amine-type N (399.7 eV, 60.42%) and amide-type N (400.5 eV, 39.58%), respectively. The XPS spectra directly confirm the presence of nitrogen in the L-CDs, along with hydrophilic functional groups such as amino groups. The high-resolution O1s spectrum displayed three peaks at 531.9 eV, 532.8 eV, and 533.7 eV, which are assigned to C=O, C−O−C, and H−O−H functional groups, respectively (Figure 3e) [27], with relative proportions of 23.10%, 41.48%, and 35.41%. The presence of H−O−H characteristic groups is attributed to water adsorbed on the surface of L-CDs. This surface hydration is consistent with the hydrophilic nature of L-CDs, which readily absorb moisture from the atmosphere. The XPS analysis results demonstrate that the surface of the L-CDs is abundant with oxygen- and nitrogen-containing functional groups, such as –COOH, –OH, −NHCO−, and –NH_2_. These findings are consistent with the FT-IR characterization results, indicating that the L-CDs prepared in this study possess excellent water solubility. This property facilitates plant absorption, making them highly suitable for applications in plant tissue culture.

### 3.2. Optical Characterization of L-CDs

**Figure 4:**
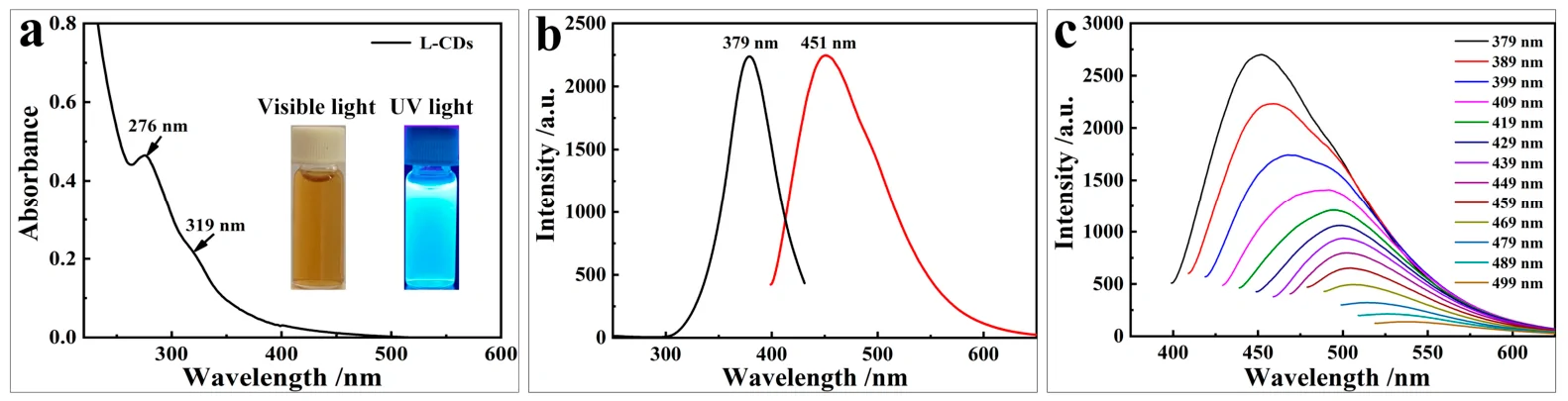
(**a**) UV-vis absorption of the L-CDs. Insets in (**a**) show the associated photographs taken under visible-light (**left**) and 365 nm UV irradiation (**right**). (**b**) PL excitation, and emission spectra of L-CDs in aqueous solution. (**c**) PL emission spectra of the L-CDs under different excitation conditions (379–499 nm).

The aqueous solution of L-CDs appeared brownish-yellow under daylight and emitted blue fluorescence under UV irradiation (inset in Figure 3a). UV-vis absorption and fluorescence spectroscopy were performed to investigate the optical properties of the L-CDs. As shown in Figure 4a, the UV-vis absorption spectrum of the L-CDs exhibited a strong characteristic absorption peak at 276 nm, which is attributed to the π-π electronic transition of C=C bonds. Additionally, a weak peak was observed at 319 nm, which is assigned to the n-π transition of C=O bonds and the defect surface states formed by nitrogen-containing structures [28]. The excitation spectrum (Figure 4b) was recorded by monitoring the emission at 451 nm. The maximum fluorescence intensity was observed at an excitation wavelength of 379 nm, which was selected as the optimal excitation wavelength for subsequent experiments, despite the stronger absorption bands observed at lower wavelengths in the UV-Vis spectrum. Using quinine sulfate in 0.1 M H_2_SO_4_ as a reference (QY = 54.0%), the fluorescence quantum yield (QY) of L-CDs in aqueous solution was determined to be 10.23%. As shown in Figure 4c, upon excitation at varying wavelengths ranging from 379 nm to 499 nm, the fluorescence emission peaks of the L-CDs exhibited a red-shift from 451 nm to 541 nm as the excitation wavelength increased. This demonstrates that the L-CDs possess a pronounced excitation-wavelength dependence. This excitation-dependent fluorescence behavior is likely attributed to the presence of surface functional groups, such as –COOH, –OH, −NHCO−, and –NH_2_, on the carbon dots. These surface states introduce new localized energy levels within the bandgap. Due to the varying energy distribution of these surface emission sites, excitation light at different wavelengths can selectively excite specific surface defect states, thereby resulting in excitation-dependent fluorescence emission. [29]. The fluorescence emission spectrum of the L-CDs shows a significant overlap with the absorption bands of chlorophyll a and b. This spectral characteristic suggests a theoretical potential for the L-CDs to act as spectral converters in agricultural applications, although further biological studies are required to verify their actual impact on photosynthesis and plant growth. This remarkable fluorescence emission property suggests that the L-CDs can serve as a light source for plant photosynthesis, thereby potentially enhancing photosynthetic efficiency and showing potential for promoting plant growth.

### 3.3. Fluorescence Stability and Biocompatibility of L-CDs

**Figure 5:**
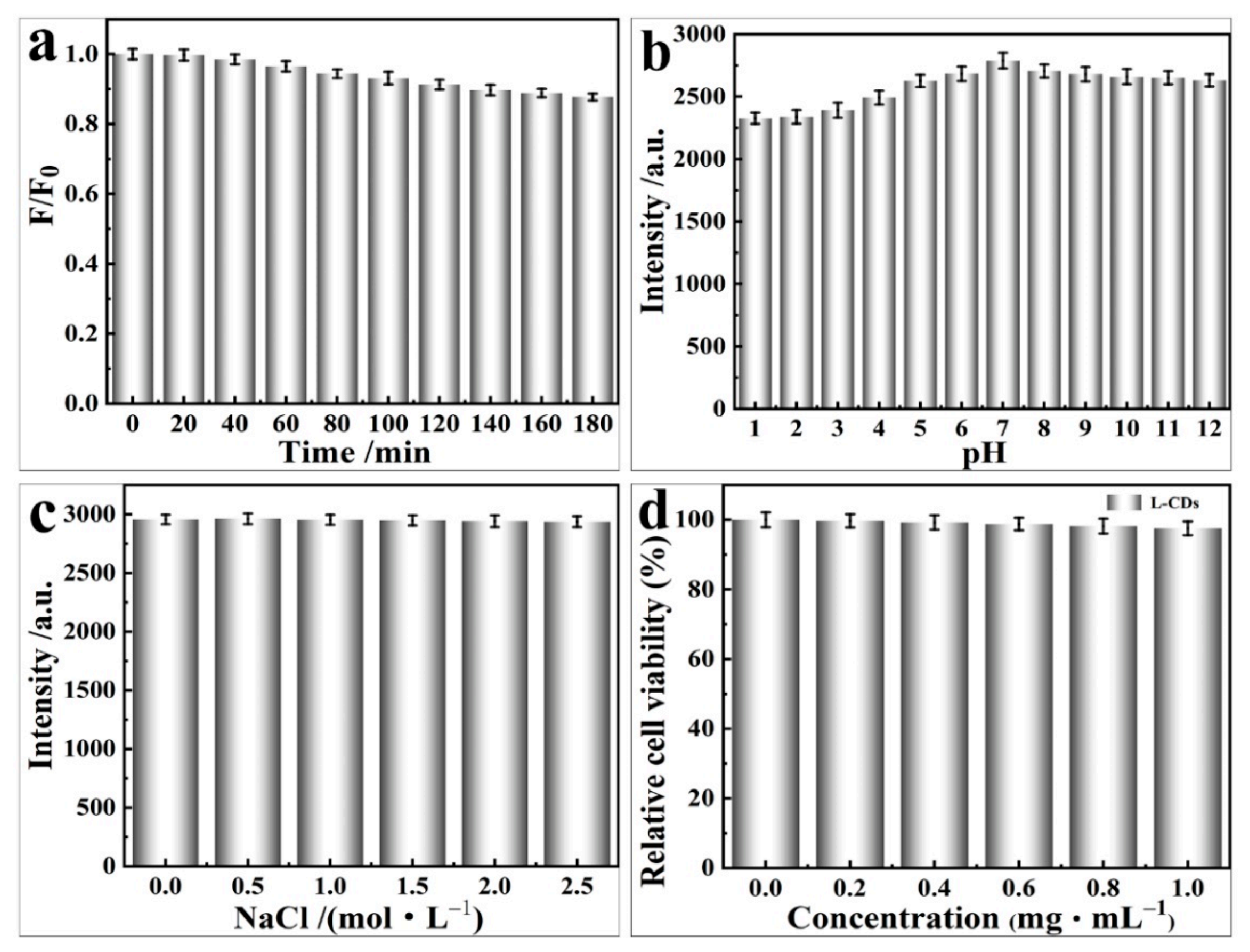
(**a**) Dependence of PL intensity on excitation time (0–180 min) for L-CDs under 365 nm. (**b**) Effect of pH on the PL intensity of L-CDs. (**c**) Effect of different concentrations of NaCl on the PL intensity of L-CDs. (**d**) Relative viabilities of L929 cells incubated with various concentrations of L-CDs (0–1 mg·mL^−1^).

Fluorescence stability is one of the critical factors determining the performance of optical materials. To investigate the fluorescence stability of L-CDs, the fluorescence intensity at the optimal emission wavelength of 451 nm was measured under the optimal excitation wavelength of 379 nm. The effects of UV irradiation time, pH, and sodium ion concentration on the fluorescence stability of L-CDs were systematically evaluated. First, the aqueous solution of L-CDs was continuously exposed to 365 nm UV excitation light for 180 min. As shown in Figure 5a, the ratio of the fluorescence intensity (F) at different irradiation times to the initial fluorescence intensity (F_0_) exhibited a fluctuation of approximately 10% over the testing period. This minor variation is attributed to [e.g., instrumental noise/slight temperature fluctuations], but the signal remained within an acceptable range, indicating good reproducibility. Therefore, the L-CDs demonstrated satisfactory stability, defined here as the ability to maintain the fluorescence intensity with a relative standard deviation (RSD) of less than 10%, indicating that the L-CDs possess excellent photobleaching resistance. Environmental pH is crucial for plant growth; therefore, the effect of pH on the L-CDs was evaluated to investigate their stability during the plant tissue culture process. As shown in Figure 5b, the fluorescence intensity of the L-CDs shown negligible variation over a wide pH range of 1−12, indicating that the L-CDs possess broad pH stability, which is highly beneficial for plant tissue culture. To study the impact of ionic strength, NaCl solutions with different concentrations were employed to simulate environmental variations. The results in Figure 5c demonstrate that the fluorescence intensity of the L-CDs remained almost unaffected as the NaCl concentration increased. This confirms that the L-CDs possess superior salt tolerance, as their fluorescence is insensitive to salt ion concentrations. This remarkable fluorescence stability is highly advantageous for their application in promoting plant growth.

The application of L-CDs in plant tissue culture necessitates rigorous evaluation of their biosafety. In this study, the biocompatibility of L-CDs was assessed using a CCK-8 cell viability assay. As shown in Figure 5d, cell viability remained almost unchanged with increasing L-CDs concentrations. Even at a concentration of 1 mg·mL^−1^, cell viability was maintained above 97%, indicating that L-CDs exhibit no significant cytotoxicity and possess excellent biosafety.

### 3.4. Effects of L-CDs on the Growth of S. grosvenorii Tissue Cultured Seedlings

**Figure 6:**
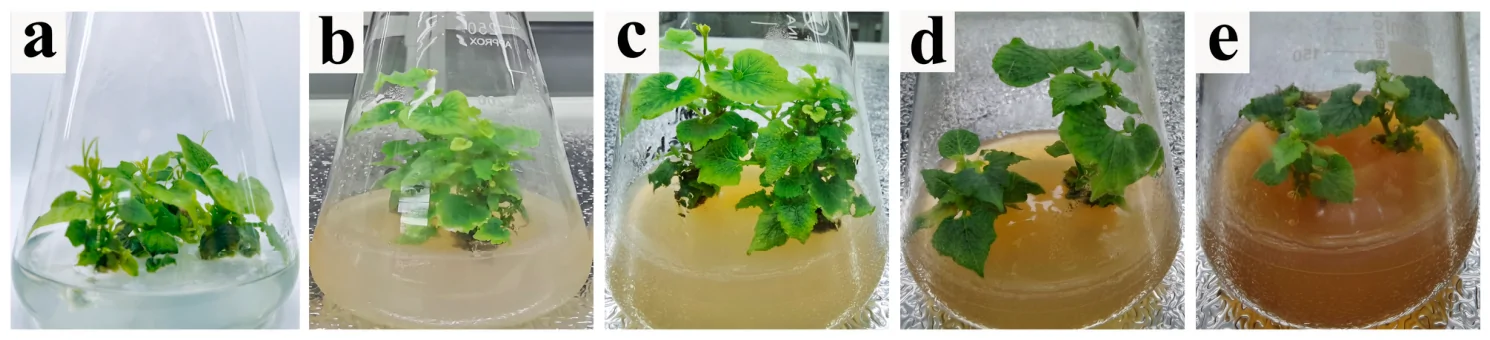
Effects of different concentrations of L-CDs ((**a**). 0, (**b**). 0.3, (**c**). 0.6, (**d**). 0.9, (**e**). 1.2 mg·L^−1^) on *S. grosvenorii* tissue culture seedlings.

Well-filled *S. grosvenorii* seeds were selected for seedling cultivation on basal MS medium without any plant growth regulators. After 7 days of inoculation, the seeds exhibited visible germination (radicle emergence). By day 20, the seedlings had reached a height of over 5 cm (Figure S1). The sterile seedlings were excised into nodal segments and inoculated onto MS medium supplemented with 1 mg·L^−1^ 6-BA and 0.2 mg·L^−1^ IBA, containing varying concentrations of L-CDs. The observations after 20 days are presented in Figure 6. At lower L-CDs concentrations, the *S. grosvenorii* plants exhibited rapid and vigorous growth, with each node producing more than two adventitious shoots (Table 1). Notably, at an L-CDs concentration of 0.6 mg/L, the *S. grosvenorii* plantlets reached an average height of 4.3 cm, with an average of 3.5 adventitious buds per plantlet. The multiplication coefficient was 4.2, and the plantlets exhibited robust growth. (Figure 6c). However, when the L-CDs concentration was increased to 1.2 mg·L^−1^, the plantlets exhibited obvious vitrification (Figure 6e) and slow growth. The average plant height was only 2.2 cm, with an average of 1.5 adventitious buds per plantlet, and the multiplication coefficient dropped to merely 1.9. In the control group, the plantlets exhibited robust growth, with an average height of 2.3 cm and an average of 2.1 adventitious buds per plantlet. The multiplication coefficient was 2.8. (Figure 6a).

### 3.5. Effects of L-CDs on Antioxidant Enzymes of S. grosvenorii Tissue Culture Seedlings

**Figure 7:**
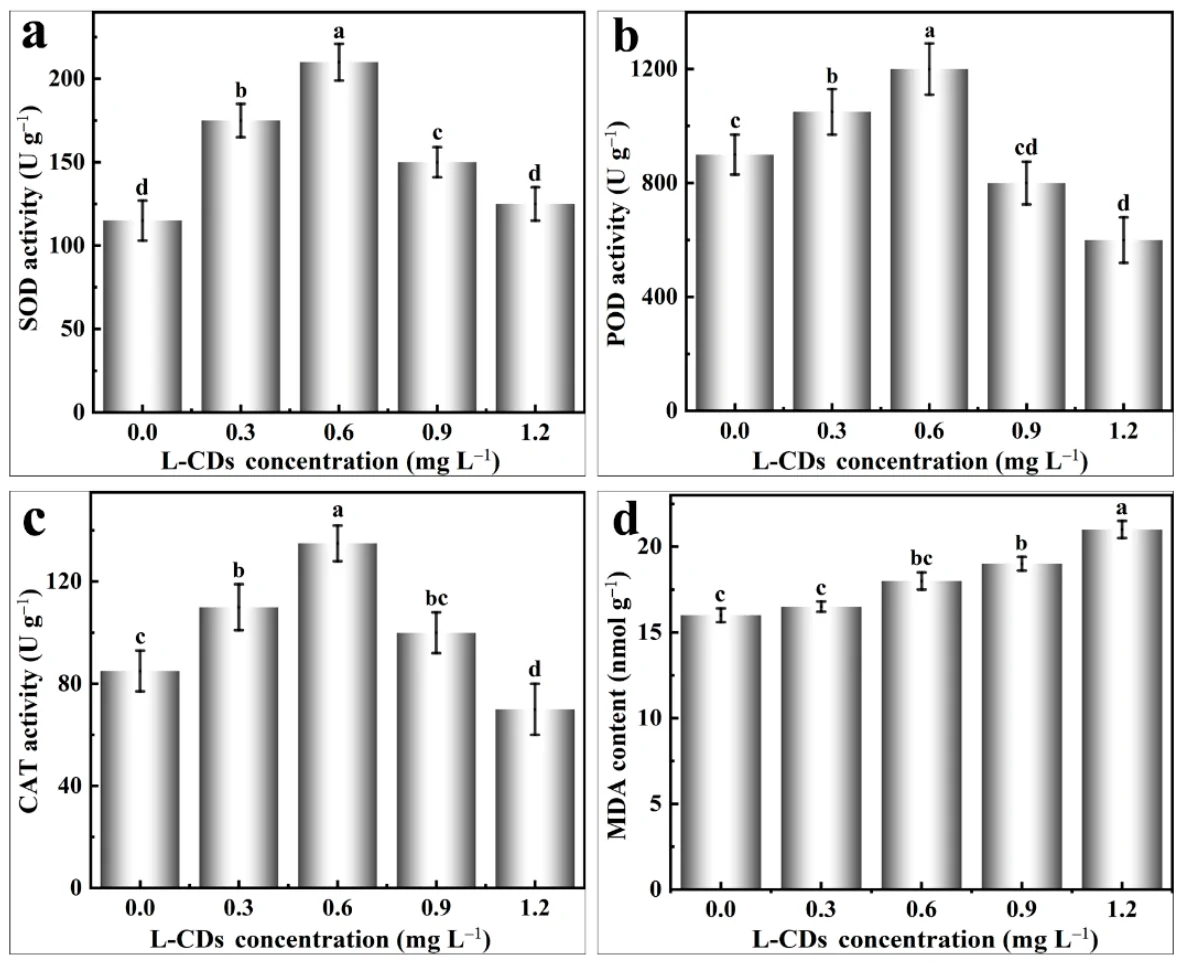
Effects of different concentrations of L-CDs treatment on SOD (**a**), POD (**b**), CAT (**c**) and MDA (**d**) in *S. grosvenorii* tissue culture seedlings. Different lowercase letters represent significant differences (*p* < 0.05).

Antioxidant enzymes serve as the first line of defense in the biological antioxidant defense system, playing a crucial role in maintaining cellular redox homeostasis and preventing oxidative damage. To investigate the effects of L-CDs on the antioxidant enzymes of *S. grosvenorii* tissue-cultured seedlings, the activities of superoxide dismutase, peroxidase, and catalase, as well as the malondialdehyde content, were determined. As shown in Figure 7, as the concentration of L-CDs increased, the activities of the antioxidant enzymes SOD, POD, and CAT in the *S. grosvenorii* tissue-cultured seedlings exhibited a trend of first increasing and then decreasing. When the L-CDs concentration reached 0.6 mg·L^−1^, the activities of SOD (Figure 7a), POD (Figure 7b), and CAT (Figure 7c) peaked, increasing by 82.61%, 33.33%, and 58.82%, respectively, compared to the control group. These results indicate that low concentrations of L-CDs can enhance the antioxidant capacity of the seedlings, helping them adapt to the in vitro growth environment. Conversely, high concentrations of L-CDs induce stress in the seedlings, which is detrimental to their growth. The MDA assay results further confirmed that the MDA content gradually increased with the rising concentration of L-CDs (Figure 7d). At an L-CDs concentration of 1.2 mg·L^−1^, the MDA level was 31.25% higher than that of the control group, indicating that the *S. grosvenorii* tissue-cultured seedlings suffered from severe oxidative stress and were in a state of stress.

### 3.6. Effect of L-CDs on the Physiological Characteristics in S. grosvenorii Tissue-Cultured Seedlings

**Figure 8:**
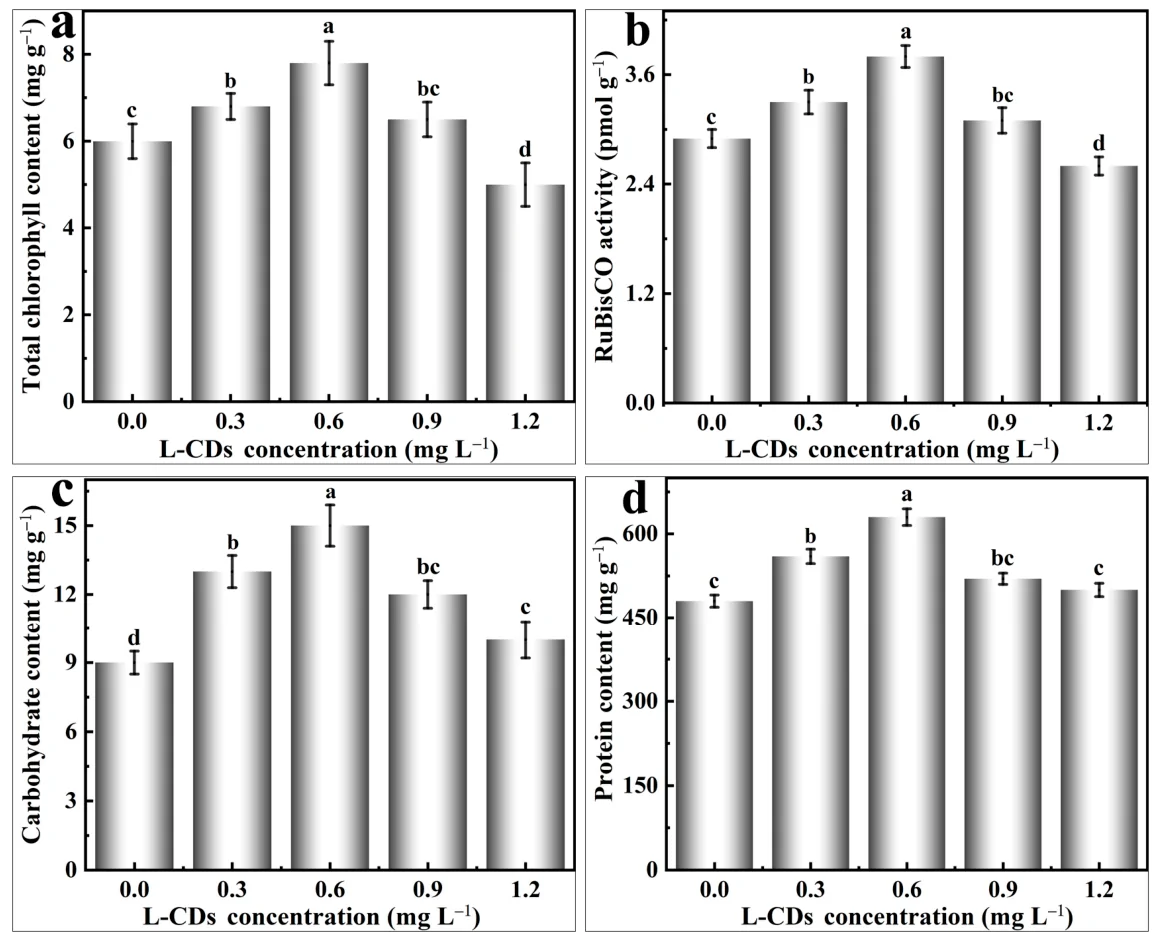
Effects of different concentrations of L-CDs treatment on total chlorophyll content (**a**), RuBisCO activity (**b**), Carbohydrate content (**c**) and Protein content (**d**) in *S. grosvenorii* tissue culture seedlings. Different lowercase letters represent significant differences (*p* < 0.05).

To investigate the effects of L-CDs on the physiological indices of *S*. *grosvenorii* plantlets, chlorophyll content, RuBisCO enzyme activity, carbohydrate content, and soluble protein content were determined. As shown in Figure 8, with the increasing concentration of L-CDs, both chlorophyll content and RuBisCO activity exhibited a trend of initially increasing and then decreasing. Since chlorophyll content and RuBisCO activity are positively correlated with photosynthetic efficiency, the highest chlorophyll content was observed at an L-CDs concentration of 0.6 mg·L^−1^, reaching 7.8 mg·g^−1^, which was 30.0% higher than that of the control (Figure 8a). Meanwhile, RuBisCO activity peaked at 3.8 pmol·g^−1^, an increase of 31.03% compared to the control (Figure 8b). These results indicate that L-CDs can enhance the photosynthetic efficiency of *S. grosvenorii* plantlets, thereby promoting their growth. Furthermore, the contents of carbohydrates and soluble proteins also reached their maximum values at 0.6 mg·L^−1^ L-CDs, showing increases of 66.67% and 31.25% over the control, respectively (Figure 8c,d). This suggests that L-CDs facilitate the accumulation of photosynthetic products, providing a solid material basis for the rapid growth of the plantlets.

## 4. Discussion

Our results demonstrate that the application of L-CDs significantly promoted the growth and increased propagation coefficient of *S. grosvenorii* in tissue culture. This finding aligns with recent studies suggesting that carbon dots can act as biostimulants in plant systems. For instance, Annmary et al. [19] reported that ACDs derived from fresh tomatoes and lemon peels Significant enhancement of callus growth in *Oncidium brassia* at a concentration of 3 mg·mL^−1^ (Table 2). Similarly, our study observed an optimal growth promotion at 0.6 mg·L^−1^. The effective dosage in our study was relatively low. This discrepancy may be attributed to differences in plant species and experimental objectives. Similar to auxin, where high concentrations favor callus induction while low concentrations promote shoot germination, the biological effects of L-CDs are highly concentration-dependent.

The L-CDs exhibited a Quantum Yield of 10.23%, indicating strong fluorescence properties. The L-CDs internalized in the plant cells can absorb UV light (which is often less utilized by plants) and down-convert it into visible blue/green light (Photosynthetically Active Radiation). This spectral shift likely enhances the light availability for chloroplasts, thereby boosting photosynthetic efficiency and providing more energy for cell division and differentiation in the tissue culture environment.

The L-CDs enhance photosynthetic efficiency by increasing chlorophyll content and RuBisCO enzyme activity. This finding is consistent with the study by Li et al. [30], who reported that Mg,N-CDs upregulated the gene expression of enzymes related to chlorophyll synthesis and metabolism in rice, thereby increasing RuBisCO activity and the contents of chlorophyll *a* and *b*, which ultimately improved photosynthetic activity.

The small size and hydrophilicity of the L-CDs enable them to penetrate the cell wall and potentially enter the vascular system. They may act as nanochannels or carriers to facilitate the transport of water and essential nutrients within the explants. This is supported by the study of Joseph et al. [31], who demonstrated that N-CDs could induce massive and compact callus formation in *Vigna radiata* L., along with a significant increase in fresh weight. This enhancement was attributed to the improved water absorption, vascular transport, hydrolytic enzyme activity, and photosynthesis mediated by the N-CDs.

The growth promotion followed a dose-dependent pattern, where high concentrations inhibited growth. This phenomenon fits the hormesis model, where low doses of nanomaterials stimulate biological systems (possibly by inducing mild stress that triggers defense and growth mechanisms), while high doses may cause cytotoxicity or block cellular transport channels.

**Table 2:** Application of carbon dots in plant tissue culture.

Sources of CDs	Synthetic Method of CDs	Plant Specie	Dosage	Effects of CDs on Plant	Mechanism of Action	Reference
Tomatoes and lemon peels	Microwave assisted	*Oncidium brassia*	3 mg·mL^−1^	Significant enhancement of callus growth	Enhanced nutrient and water uptake, Photosynthesis enhancement, Cell division stimulation	[19]
Sauropus androgynus leaves, Ammonia	hydrothermal	*Vigna radiata* L.	1.8 mg·mL^−1^	Generate profuse, dense callus with extensive rooting, significantly increased fresh weight	Enhanced water imbibition, vascular translocation, enhanced hydrolytic enzyme activity, Photosynthesis enhancement	[31]
High-purity graphite rods (99.99%), Polyethyleneimine	electrochemical etching	Rice, Wheat, Mung bean	/	Gene delivery	Size-dependent cell wall penetration,	[32]
This word	hydrothermal	*Siraitia grosvenorii*	0.6 mg·L^−1^	Significantly promoted the growth, increased propagation coefficient,	Increase chlorophyll content and enhance RuBisCO activity, enhanced antioxidant enzyme activities	

## 5. Conclusions

In summary, L-CDs were synthesized via a one-step hydrothermal method using *S. grosvenorii* leaves as the carbon source. The morphology, composition, and structure of the L-CDs were systematically investigated using TEM, XRD, Raman, FTIR, and XPS techniques, while their optical properties were explored via UV-vis absorption and fluorescence spectroscopy. Furthermore, CCK-8 cell viability assays confirmed the excellent biocompatibility of the L-CDs. Subsequent studies on *S. grosvenorii* tissue-cultured seedlings revealed that low concentrations of L-CDs promote seedling growth and enhance antioxidant enzyme activities, whereas high concentrations are detrimental to their growth. Overall, this study expands the application potential of CDs, providing a novel approach for plant breeding and propagation, as well as new avenues for agricultural development.

## Figures and Tables

**Table 1:** Effects of L-CDs on *Siraitia grosvenorii* tissue-cultured seedlings.

L-CDs Concentration (mg·L^−1^)	Plant Height (cm)	Number of Adventitious Buds	Multiplication Coefficient	Growth of Adventitious Buds
0	2.3 ± 1.2 c	2.1 ± 1.2 c	2.8 ± 1.1 c	Slow growth, small leaves, and tender yellow coloration.
0.3	3.5 ± 1.2 b	2.7 ± 1.2 b	3.6 ± 1.3 b	Faster growth, larger leaves, and yellowish-green coloration.
0.6	4.3 ± 1.5 a	3.5 ± 1.2 a	4.2 ± 1.5 a	Faster growth, larger leaves, and vibrant green coloration.
0.9	3.2 ± 2.1 bc	2.3 ± 1.2 c	3.3 ± 1.4 c	Faster growth, larger leaves, and vibrant green coloration.
1.2	2.2 ± 1.3 c	1.5 ± 0.6 d	1.9 ± 1.0 d	Slow growth, small leaves, and vitrification.

Note: The culture medium is MS + 6-BA 1 mg·L^−1^ + IBA 0.2 mg·L^−1^; Multiplication coefficient = Total number of proliferated plantlets/Total number of inoculated plantlets. Different lowercase letters after data in the same column indicate significant differences (*p* < 0.05).

## Data Availability

The data presented in this research are available on request from the corresponding author.

## Supplementary Materials

The following supporting information can be downloaded at: https://www.mdpi.com/article/10.3390/nano16181185/s1, Figure S1. Tissue culture seedlings of *Siraitia grosvenorii*.
