# Supplementary material for: Preparation of Siraitia grosvenorii Leaf-Derived Carbon Dots and Its Effect on Tissue Culture Seedlings of Siraitia grosvenorii
Source: Nanomaterials (Basel). 2026 Sep 20;16(18):1185. doi: 10.3390/nano16181185 (PMC13610279; doi:10.3390/nano16181185)
Supplement: Supplementary file 1 [file nanomaterials-16-01185-s001.zip › nanomaterials-4536462-supplementary.pdf]

Supporting Information

Preparation of *Siraitia grosvenorii* Leaf-Derived Carbon Dots and Its Effect on Tissue Culture Seedlings of *Siraitia grosvenorii*

Lizhen Zhang [1], Yihan Liu [2,*], Dongye Yang [3,*] and Jiayue Deng [1]

[1] School of Chemistry and Life Health, Guilin Normal College, Guilin 541119, China; zhanglizhen@mail.glnc.edu.cn (L.Z.)

[2] Suzhou Institute of Pharmaceutical Innovation, Suzhou Industrial Park, Suzhou 215218, China

[3] Scientific Research Center, Guilin Medical University, Guilin 541000, China

* Correspondence: mc25502@connect.um.edu.mo (Y.L.); 112005046@glmc.edu.cn (D.Y.)

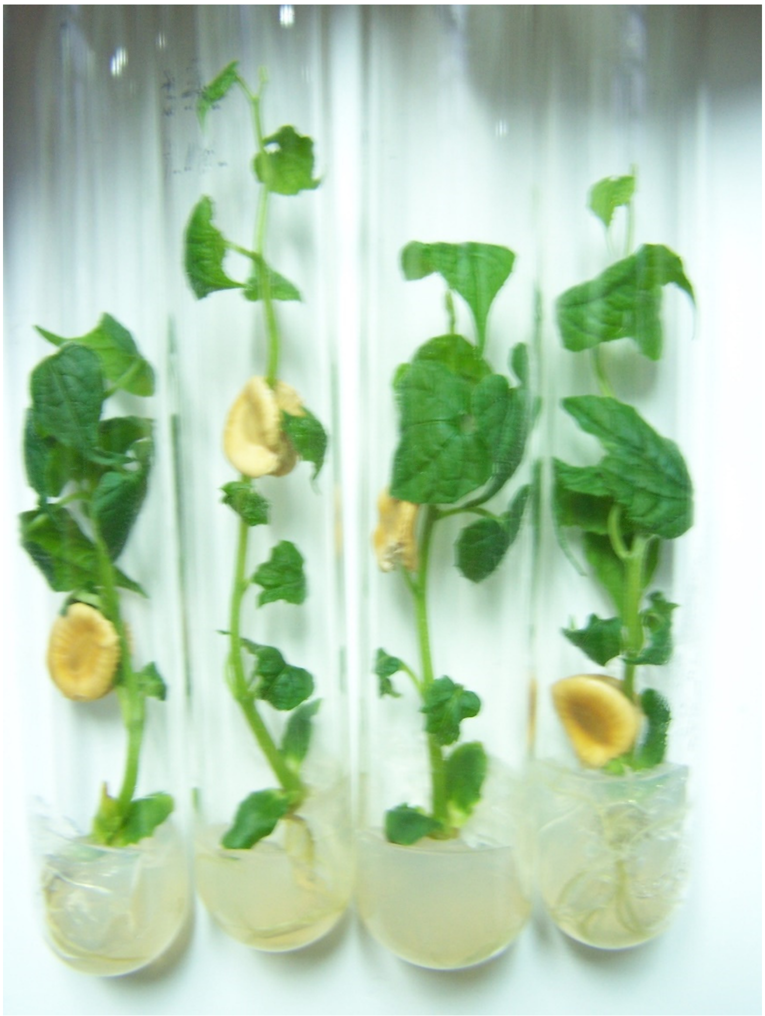

Figure S1. Tissue culture seedlings of *Siraitia grosvenorii*
